# Concise practice recommendations for the provision of andrological services and assisted reproductive technology for male infertility patients during the SARS-CoV-2 in Brazil

**DOI:** 10.1590/S1677-5538.IBJU.2020.06.03

**Published:** 2020-09-02

**Authors:** Jorge Hallak, Sandro C. Esteves

**Affiliations:** 1 Centro de Ciência e Inovação em Andrologia Clínica e Laboratório de Alta Complexidade em Saúde Reprodutiva e Sexual Masculina São PauloSP Brasil ANDROSCIENCE, Centro de Ciência e Inovação em Andrologia, Clínica e Laboratório de Alta Complexidade em Saúde Reprodutiva e Sexual Masculina, São Paulo, SP, Brasil;; 2 Universidade de São Paulo SP Brasil Disciplina de Urologia, Universidade de São Paulo, SP, Brasil;; 3 Grupo de Estudos em Saúde Masculina Instituto de Estudos Avançados Universidade de São Paulo SP Brasil Grupo de Estudos em Saúde Masculina, Instituto de Estudos Avançados, Universidade de São Paulo, SP, Brasil;; 4 Unidade de Toxicologia Reprodutiva Departamento de Patologia Universidade de São Paulo SP Brasil Unidade de Toxicologia Reprodutiva, Departamento de Patologia, Universidade de São Paulo, SP, Brasil;; 5 Clínica de Andrologia e Reprodução Humana CampinasSP Brasil ANDROFERT, Clínica de Andrologia e Reprodução Humana, Campinas, SP, Brasil;; 6 Departamento de Cirurgia Universidade Estadual de Campinas CampinasSP Brasil Departamento de Cirurgia, Disciplina de Urologia, Universidade Estadual de Campinas (UNICAMP), Campinas, SP, Brasil;; 7 Faculdade de Saúde Universidade de Aarhus Aarhus Dinamarca Faculdade de Saúde, Universidade de Aarhus, Aarhus, Dinamarca

## INTRODUCTION

The pandemic caused by the severe acute respiratory syndrome coronavirus 2 (SARS-CoV-2), responsible for the disease so-called COVID-19, represents the most exceptional health, social, economic, and humanitarian crisis known to humankind since the H1N1 flu of 1918. So far, it affected 213 countries, infecting over 100,000 people daily worldwide, with hundreds of thousands of deaths ( 2 , 3 ). Restrictions to personal freedom and partial or complete lockdowns have been implemented to safeguard public health, with a noticeable impact on urological practice. Currently, diagnostic semen analysis, sperm banking in non-oncological patients, elective surgical sperm retrieval (SR), and related fertility procedures are rated as of low priority in most countries due to the COVID-19 pandemic ( 4 ). Based on expert best judgment, regulatory authorities, urological, and reproductive medicine societies have considered that postponing care in the above scenarios for six months or longer will have an unlikely risk of clinical harm. However, it has been argued that the pandemic may last many months, even years, and experts believe of second and third waves in the months to come. It is therefore evident that in the absence of a vaccine or broad herd immunity, not only urgent short-term responses but also long-term measures are essential in this most uncertain time.

### Which male infertility patients to prioritize for the continuation of care delivery?

Recently, a group of 27 experts from 15 countries and five continents has argued that postponing andrological services and male infertility care during the COVID-19 pandemic could permanently compromise the prospects of biological parenthood for ‘time-sensitive’ patients, thus resulting in a devastating psychological impact on men undergoing fertility-related treatment ( 1 ).

**Figure 1:**
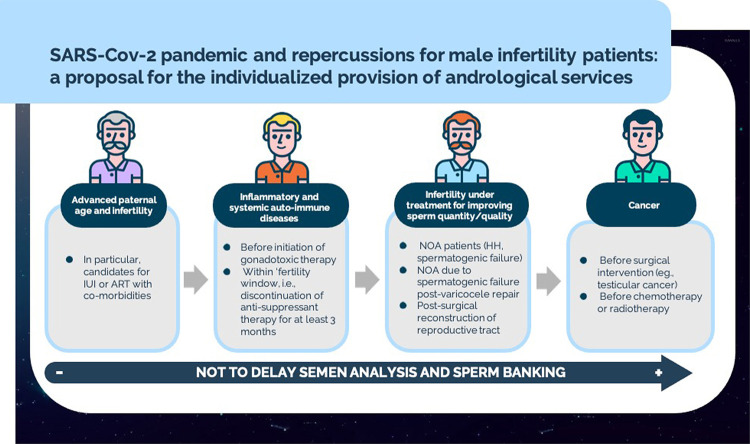
SARS-CoV-2 pandemic and provision of andrological services: proposal for individualized management.

The group utilized the term ‘time-sensitive’ to categorize patients in whom the fertility ‘window’ may be transitory, as listed below ( Figure-1 ).

1. Severe male infertility (e.g., azoospermic/cryptozoospermic men under medical or post-surgical treatment to improve sperm quantity/quality);
2. Inflammatory and systemic autoimmune diseases;
3. Cancer, chemotherapy, radiation or immunosuppressive therapy;
4. Advanced paternal age (e.g., >50 years).
5. Practice recommendations for safe care delivery.

An evidence-based analysis was conducted concerning the reasons why these patients should be prioritized for care delivery during the pandemic. Moreover, the group of experts developed a series of practical recommendations on how to most optimally provide care in a safe environment, considering patients, staff, and the community as a whole ( Table-1 ). The intended goals were to, first of all, alleviate the adverse impact of the SARS-Cov-2 pandemic in the months to come, thus offering reproductive urologists and patients alike greater autonomy, and secondly, help authorities and healthcare providers identify which male infertility patients should be prioritized during the SARS-CoV-2 pandemic for the continuation of care in a safe environment.

**Table 1:** Practice recommendations for safe delivery of care to male infertility patients during the SARS-CoV-2 pandemic* in Brazil

Recommendation	Additional remarks
1. Before any service is provided, active SARS-CoV-2 infections and suspected cases should be excluded.	Patient testing should include RT-PCR and/or blood antibody, preferably using ELISA. Ideally, only samples from patients with negative PCR results or who have acquired IgG antibodies through past infection or herd immunity should be treated or have sperm cryopreserved.
2. Andrological services (e.g., diagnostic semen analysis, sperm functional tests and sperm cryopreservation) should not only be available for oncological patients, but also for the group of ‘time-sensitive’ patients.	i. Severe male infertility under medical or surgical treatment aiming at improving sperm quantity or quality (e.g., patients with non-obstructive azoospermia (NOA) or cryptozoospermic/severe oligozoospermia, including post-varicocele repair, and those with evidence of loss of patency after successful surgical reconstruction of the reproductive tract). ii. Men at reproductive age affected by inflammatory diseases or systemic inflammatory diseases (SADs), i.e., before initiation of gonadotoxic therapy or if under the ‘fertility window’ achieved after temporary (at least three months) discontinuation of therapy. iii. Infertile men older than 50 years, in particular those with comorbidities who are candidates for intrauterine insemination (IUI) or assisted reproductive technology (ART), and who are concerned about the risk of acquiring SARS-CoV-2 and/or the possibility of using anti-viral therapy with possible gonadotoxic effects.
3. Surgical sperm retrieval and cryopreservation of testicular sperm or testicular tissue should be considered in specific situations involving men with NOA undergoing medical therapy to improve spermatogenesis.	In this setting, procedures should be performed only for PCR negative or IgG positive patients. The use of electrocautery should be used with caution or used with an air-aspiration negative pressure device; the surgical smoke might carry the virus in case a patient is infected but asymptomatic, despite a negative testing. Only essential staff should stay in the operating theater, and personal protection measures should be strictly followed as determined by the local healthcare authorities. In closed-controlled air systems, the airflow might produce an increase in the viral spread from potential asymptomatic patients. Thus, special attention should be given to air quality control, including the use of air filtration systems, particularly in surgical and laboratory areas.
4. Encourage telemedicine and phone counseling for providing instructions about testing and sperm banking.	None.
5. Adherence to infection prevention recommendations is of utmost importance for patients and health practitioners alike.	This advice includes the use of appropriate personal protective equipment (PPE) by healthcare staff, adherence to social distancing measures for healthcare staff and patients, and space out appointments so that no patients are waiting together in the clinic waiting area. The importance of training staff (receptionists, nurses, technicians, doctors) on PPE needs and usage is highlighted (https://www.cdc.gov/coronavirus/2019-ncov/hcp/clinic-preparedness.html).
6. The precautionary principle and good laboratory practices should be strictly applied when handling the seminal fluid by in the andrology and embryology laboratories.	This advice includes (i) use of class II safety cabinets, which gives protection to the specimen handled as well as the operator performing the work, (ii) use of high-security vials for sperm cryopreservation, as routinely recommended by certified andrology labs and sperm banks, and (iii) additional measures to protect the specimens from laboratory staff (e.g., use of googles, N95 mask, gown/coverall, and gloves) –who might be infected but asymptomatic for SARS-CoV-2.
7. Technicians/biologists should, ideally, be tested by RT-PCR and/or ELISA blood antibody testing before resuming activities, and only staff with negative results or who became IgG positive or have acquired herd immunity should perform laboratory duties.	Although the risk is minimal, if the staff that manipulated specimens get infected during the pandemic and is actively working in the lab, an aliquot of cryopreserved semen samples should be tested (e.g., by RT-PCR) because semen samples, cryopreservation media, cryovials, and pipette tips could be contaminated by asymptomatic PCR-positive biologists and technicians.
8. A thorough discussion between patients and healthcare providers should be made for responsible shared decisions.	This advice includes the development and use of dedicated informed consent, detailing the risks of attending the facility and being treated or banking of sperm during the SARS-CoV-2 pandemic. Furthermore, explanatory material should be made available for patients. Psychological support and financial aid might be offered to those in need. The latter might be particularly relevant to patients under economic pressure due to the pandemic who need to afford the costs of semen analysis, sperm functional tests and sperm banking.
9. Advanced planning should guide the continuation of andrological services.	Working groups and quality managers should determine which patients to prioritize and how working lists should be filled, including staff scheduling. A multi-professional group including reproductive urologists, andrologists, technicians and other relevant health care professionals should work together to provide the optimal care in a safe environment.

* Esteves SC, Lombardo F, Garrido N, et al. SARS-CoV-2 pandemic and repercussions for male infertility patients: A proposal for the individualized provision of andrological services [published online ahead of print, 2020 May 1]. Andrology. 2020;10.1111/andr.12809. doi:10.1111/andr.12809

RT-PCR: Reverse-Transcriptase Polymerase Chain Reaction; ELISA: Enzyme Linked Immuno Sorbent Assay; IgG: Immunoglobulin class G

### The Brazilian scenario

Brazil has become the new epicenter of the SARS-CoV-2 pandemic, with over 2.5 million confirmed cases making it the second country in absolute number of deaths after the United States. The death toll is growing steadily, with a significant number of daily deaths in the lower four figures. Experts believe the situation can deteriorate further due to the typical economic constraints in our country, in particular, if containment measures, including the widespread use of facial masks, and social distancing do not work properly. A recent probabilistic pilot study conducted in seven districts of the city of São Paulo to estimate the prevalence of herd immunity showed that about 5.2% individuals had SARS-CoV-2 IgG antibodies, corresponding to an overall infection rate of 5.192 per 100,000 individuals. Importantly, the number of infected individuals in the population has been estimated to be 11.9 times higher than the number of confirmed cases by epidemiological vigilance authorities, meaning that for each individual that tests positive for SARS-CoV-2, another 12 individuals have been infected without knowing it, with evident implications for viral spreading dynamics ( 5 ).

Along these lines, the first National Study estimating the percentage of infected people in Brazil found that the number of individuals presenting with IgG antibodies for COVID-19, irrespective of developing COVID-19 clinical manifestations, was seven times higher than reported. If these projections were applied to the entire country, the number of infections would skyrocket to something between 10 and 12 million cases. Unfortunately, at present, only about 2% of the entire population have been tested. Moreover, only 1.4% of the 25,025 tested individuals in 90 cities across the country has developed IgG antibodies, thus indicating that we have much to cover before the estimated 82% population needed to be infected for herd immunity in a disease that has a reproduction number (R0) around 5.7, that is, each infected individual has the potential to spread the disease to another 5 to 6 individuals ( 6 , 7 ). On the other hand, these figures suggest that the quarantine measures might be working, as shown by a recent study that tested 700 individuals in a medium-sized city with high-income under a 70-day quarantine ( 8 ). In this study, the infected ratio was coincidentally 1.2%.

The global mortality rate is around 4.7%, similar to that reported in Brazil ( 3 ). One aspect of COVID-19 in our continental country is its unequal impact in different urban settlements. The number of deaths is higher in peripheral city-areas and in regions where the economic crisis had already reached before the pandemic, regions with not enough or inexistent intensive care units, neither enough artificial breathing apparatus nor highly qualified medical staff. On the one hand, for each death notified by health authorities, another 1.67 to 2.72 occur without testing and notification; therefore, the number of deaths estimated by scientific methods of “nowcasting” should be around 1.5 times higher than officially reported ( 7 , 9 ). On the other hand, Brazil faces a shortage of tests due to a worldwide shortage of laboratory supplies.

Consequently, mainly health professionals in the frontline and symptomatic patients have been widely tested. Therefore, the 4.1% death rate seems to be unrealistic, because if one divides the number of deaths by a more significant number of tested individuals, the actual death rates go proportionally down. The pilot study conducted in the city of São Paulo calculated an overall death rate of around 0.95% ( 5 ).

The overwhelming majority of Brazilian fertility centers, andrology laboratories, and sperm banks are located in major cities, most of which have been profoundly affected by the SARS-CoV-2 pandemic. Also, reproductive urologists are clustered in large cities. Thus, infertility patients living in these cities or traveling from other areas to attend these facilities are at risk of getting infected. Although the reported morbidity rate in patients at reproductive age is low, there is a real risk of patients and health care providers to spread the infection in the context of asymptomatic viral shedding. Moreover, infected men present an age-independent higher risk for adverse outcomes and death ( 10 ). These observations are probably related to the fact that men have more chronic diseases than women of the same age, including obesity, hypertension, heart conditions, diabetes, and metabolic syndrome, all linked to higher severity of manifestations ( 10 ). It is well known that male infertility is associated with a diverse range of general medical conditions, including hypertension ( 11 ), obesity ( 12 ), ischemic heart disease, diabetes ( 13 ), and a broad range of cancers ( 14 ). Thus, this population deserves special attention. While it is critical for reproductive urologists and fertility centers to continue to offer care to this vulnerable population, it is also essential that all efforts are undertaken to deliver services in a safe environment ( 15 ). Equally important is to discuss the possible adverse impact of SARS-CoV-2 on male reproductive health.

### SARS-CoV-2 and the genitourinary tract

The “cytokine storm” seems to be a central element in the pathophysiology of severe COVID-19. It relates to an excessive and uncontrolled release of pro-inflammatory agents, commonly seen in graft-versus-host situations, multiple sclerosis, pancreatitis, and multiple organ failure. Recent studies suggest that cellular and molecular mechanisms contribute to the cytokine storm in viral diseases (e.g., influenza and other respiratory viruses, including SARS-CoV-1). However, its severity is overwhelmingly higher in SARS-CoV-2 because of the mechanisms involved in viral and cell membrane fusion. Cytokines are a group of proteins (e.g., interferons, interleukins, chemokines, tumor-necrosis [TNF], colony-stimulating factors) used for intercellular signaling and communication, with autocrine, paracrine, and endocrine activity, having the capacity of launching a wide array of intracellular responses through receptor-binding in specific target cells ( 16 ).

The semen of healthy men contains a variety of cytokines with immunologic roles, including the regulation of T-cell response and macrophage activity. They are involved in immune defense against genital infections; however, elevated levels of interleukin-1, 2 and 6 and TNF-alpha have been associated with defective steroidogenesis, poor semen quality, and male infertility ( 17 - 19 ). Thus, these molecules work as protectors of normal testicular function when released in limited and controlled physiological conditions. However, that balance is quickly transformed into a deleterious effect if higher amounts of many classes of cytokines, including the transforming growth factor (TGF) superfamily, are released ( 17 ).

A critical aspect of the SARS-CoV-2 infection for male reproductive health relates to the virus cell-attaching mechanism. Angiotensin-converting enzyme 2 (ACE2) belongs to the angiotensin-converting enzyme family of dipeptidyl carboxypeptidases, which is homologous to human angiotensin 1 converting enzyme. ACE2 is a functional receptor for SARS-CoV-2 ( 19 ). A remarkable finding is that the testes are ranked second in the number of ACE2 receptors after the lungs. SARS-CoV-2 enters the cell through ACE2 receptors via the spike-protein (S), which facilitates viral attachments to the surface of target cells after priming by cellular proteases, in particular, transmembrane protease serine 2 (TMPRSS2). The ‘S’ protein locates in the virus’s external surface and interfaces with the cell by using a receptor-binding domain. This way, the virus can survive by completing intracellular replication and then releasing more viruses that will infect other cells and tissues. In parallel, this process produces cytotoxicity, meaning that organs with higher viral load and ACE2 receptors with TMPRSS2 expression are more prone to be heavily affected (e.g., kidneys and testes).

ACE2 receptors are also found in other reproductive structures and the bladder. Although only a few reports found that orchitis might be a consequence of both SARS-CoV 1 and 2, initial autopsies of few patients who died from SARS-CoV-1 in China revealed a clear pattern of widespread germ cell destruction, few or no spermatozoon in the seminiferous tubules, thickened basement membrane, and leukocyte infiltration ( 20 ). Moreover, the numbers of CD3þ T lymphocytes and CD68þ macrophages were significantly increased in the interstitial tissue compared with controls. The downside of this first publication it that no SARS viral genomic sequence was detected in none of the testes analyzed by in situ hybridization. However, immunohistochemistry demonstrated abundant IgG precipitation in the seminiferous epithelium of SARS testes, suggesting possible immune response as the cause for the damage, but without real proof-of-concept ( 20 ). Nevertheless, Duarte-Neto and co-workers, in a recent paper using histology and RT-PCR for SARS-CoV-2, identified systemic-organ involvement, including the lungs and testes ( 21 ). In this report, two patients developed orchitis, confirmed by ultrasound-guided minimally invasive autopsy (MIA-US).

In another study, Pan et al. also reported signs suggestive of orchitis in 16% of SARS-Cov-2 infected patients ( 22 ). In their cohort of 34 patients, however, RT-PCR carried out 30 days after disease recovery did not detect the virus in the semen. By contrast, in a study involving 38 subjects with either acute infections or after recovery, Li et al. found the virus in the semen in 15.8% of individuals ( 23 ). While many questions remain concerning the real risk of virus presence in the seminal fluid, it seems prudent to exercise the utmost precautions for sperm handling during diagnostic testing (e.g., semen analysis) and treatments involving sperm handling, including intrauterine insemination (IUI), assisted reproductive technology (ART), and cryobanking.

Most enveloped RNA viruses remain viable in ultra-low temperature (e.g., influenza), and although the risk of cross-contamination is minimal, practitioners should apply the precautionary principle ( 24 , 25 ). It is important to stress that liquid nitrogen cannot kill the coronavirus; therefore, all efforts should be made to avoid contamination. The responsible use of ART, which should drive the medical practice in this field, is even more critical at this challenging time to safeguard the health of the couple seeking fertility, the offspring, and future generations ( 26 ).

Given the observations above and the fact that the ACE2 receptor is essential for spermatogenesis, sperm maturation, and spermiation in physiological conditions ( 27 ), it seems sound to assume that the SARS-CoV-2 poses a threat for the male reproductive health. Thus, further research is warranted to understand its implications to the male reproductive health as every organ/tissue/cell that expresses ACE2 receptors, and the serine protease TMPRSS2, is a potential target for viral invasion, including the testis ( 28 ), the Leydig cell ( 29 ), the spermatozoa ( 27 ) and eventually cells dispersed in the seminal fluid ( 23 ).

## CONCLUSIONS

We reiterate that andrological services and male infertility care cannot be considered low priority during the current SARS-CoV-2 pandemic, particularly for the most vulnerable patients, like those with cancer, patients using immunosuppressive therapy, and the azoospermic/cryptozoospermic men under medical or post-surgical treatment to improve spermatogenesis. Postponing care to ‘time-sensitive’ male infertility patients could permanently compromise the prospects of biological parenthood. However, care should be provided in a safe environment for patients, staff, and community, and we, therefore, not only reaffirm the value of the recently issued practice recommendations but also firmly believe they should be strictly applied in Brazilian healthcare facilities that have restarted-or are planning to restart- delivery of relevant services. Dedicated certified andrology laboratories are best prepared to deliver both diagnostic and therapeutic services in a safe environment -during an emergency like the current one and also at normal times. We should continue to look critically and objectively at the rapidly growing SARS-CoV-2 evidence and investigate the implications of the virus to male reproductive and sexual health.
